# Supplementary material for: Are carbon emissions trading and green financial instruments synergistic? -Comprehensive quantitative research based on content analysis
Source: PLoS One. 2024 Mar 7;19(3):e0298601. doi: 10.1371/journal.pone.0298601 (PMC10919601; doi:10.1371/journal.pone.0298601)
Supplement: S1 Data — (ZIP) [file pone.0298601.s001.zip › Data/地级市Z_F工作报告.zip!/║╙▒▒/▒ú╢¿╩╨/└┤╫╘╣1⁄2╓┌║┼í╛┬φ┐╦╩2╛▌═°í┐_2008.docx]

各位代表：

现在，我代表保定市人民政府向大会报告工作，请予审议，并请市政协各位委员和列席会议的同志提出意见。

一、2008年工作回顾

过去的一年，是极不平凡的一年。面对复杂的经济形势和一系列重大挑战，在省委、省政府和中共保定市委的正确领导下，我们紧紧依靠和团结带领全市人民，全面落实科学发展观，围绕又好又快发展、强市兴县富民这个中心任务，坚持加快发展、安全发展两手抓，力保“经济不下滑、稳定不出事”，较好地完成了市十三届人大一次会议确定的各项任务。

过去的一年，我们与伟大祖国同呼吸、共命运，经受了多方面严峻考验。面对南方罕见的冰雪灾害，我市迅速组织电力职工赶赴灾区支援抢险，捐助的变压器、电缆等物资在第一时间发往灾区。面对震惊世界的汶川特大地震，我们立即组织医疗、抢险、通讯、运输等近千人的队伍前往救援。全市人民情系灾区，踊跃捐款捐物3亿多元，走在全省前列。援建安置房2735套，提前超额完成任务，受到建设部表扬。面对举世瞩目的北京奥运会，我们站在维护国家形象和民族荣誉的高度，全力保障平安奥运、绿色奥运。按照上级要求关停3000多家企业，组织近100万人参与，50多万人上一线昼夜严防死守，各级财政投入资金4亿多元。在历时百天的时间里，全市广大干部群众尽了最大努力，实现了“四个坚决防止”[1]的目标，中央领导给予充分肯定。可以说，奥运会的成功举办，保定人民做出了重要贡献！面对突如其来的三鹿奶粉事件，作为婴幼儿近百万的人口大市、奶牛存栏20多万头的畜牧大市，我们紧急行动，确定近40家定点医院，组织30多支医疗队伍入乡进村，全力筛查救治患儿。出动执法人员4万多人次，检查经营户6万户次，销毁问题乳制品300多吨，整顿奶站近300个。各级财政补贴资金4700多万元，努力把奶农和企业损失降到最低。全省现场会在我市召开，中央和省领导给予高度评价。面对百年不遇的世界金融危机，我们抢抓国家扩大内需机遇，分六条战线对接项目，首批争得2.5亿元资金、近200个项目，在全省名列前茅。同时组织力量深入企业，想方设法“驱寒送暖”过难关。回顾这一年，我们经历了太多的艰辛和感动。至今历历在目的是，抗震救灾中男女老幼排队捐款献血的场面，奥运安保中一线干警深夜排查车辆的身影，三鹿奶粉事件中医护人员废寝忘食为患儿检查的情景……，所有这些，无不体现了保定人民顾全大局、无私奉献的精神境界，体现了全市上下团结一心、众志成城的强大力量，让我们由衷地感到自豪和骄傲！

过去的一年，我们面对严峻挑战，全力推动经济平稳较快增长。在国内外经济环境突变、安全稳定压力巨大的情况下，我们始终咬定发展不放松，继续坚持“打基础、增后劲”不动摇，千方百计加快发展。狠抓农业生产和农村基础设施建设，粮食连续6年增产，第一产业增加值增长6.5%，高于全省平均增速近2个百分点。加强经济运行监测和调节，支持重点企业，培育重点产业，规模以上工业增加值增长16.9%，增速全省第一，比上年前移6位。坚持内外贸易一齐抓，社会消费品零售总额增长21.7%，比上年提高6个百分点；出口创汇增长40.5%。努力克服土地、资金等瓶颈制约，集中资源要素，狠抓项目建设，争取项目用地指标是省下达计划的2倍多，居全省前列；省挂账重点项目141个，总投资1996亿元，项目数量全省第二，投资额全省第三，完成投资计划全省第二；全市城镇固定资产投资完成713亿元，增长34.8%，增速全省第三，比上年前移6位。积极推进重点领域改革，依棉等骨干企业战略重组取得新突破。组建了3个专业招商中心，大规模开展招商引资活动，外商直接投资5.3亿美元，增长两倍多，总量、增速全省第二。我市与清华大学合作研究制定了国内外第一个《低碳城市发展规划》，出台了《关于建设低碳城市的意见》。强力推进“双三十”、“8+13”[2]重点工程建设，所有县（市）规划建设的污水处理厂全部开工，万元生产总值综合能耗下降5%，化学需氧量、二氧化硫排放量完成省定目标。全市生产总值完成1581亿元，增长11.7%，高于全省平均水平1.6个百分点。全部财政收入149.5亿元，增长22.6%，其中地方一般预算收入66.6亿元，增长29.4%，增速全省第一，比上年前移9位。城镇居民人均可支配收入12312 元、农民人均纯收入4331元，分别增长12.7%和9%。可以说，在国内外经济出现严重困难的情况下，我市经济增长稳健，主要指标在全省排位上升，表现出一定的抗御风险能力。

过去的一年，我们强力推进城镇面貌“三年大变样”，拉开了大规模城市建设序幕。把推进“三年大变样”作为加快城镇化的主要抓手，作为扩内需、保增长的引擎，作为改善民生、改善环境的重要内容，围绕开通大水系、构建大交通、建设大城市，市级领导分包重点项目，各级干部一线指挥参战，广大市民积极配合，取得了阶段性成果。市区累计拆除违、临、破、旧、陋“五拆”建筑近400万平方米、实体围墙239处。启动城中村改造、棚户区改建、旧小区改善“三改”项目28个。实施40项重点工程，其中七一路高速引线改造即将完工，五四路改造、朝阳大街景观整治初见成效，东三环建设全面启动，光园、西大街等古迹修复工程正加紧实施，全省最大的城市互通立交桥——电谷立交桥工程过半。百万市民受益的王快·西大洋两库连通工程开工建设。贯穿山区6县的张石高速一期、廊涿高速保定段竣工通车，张涿高速开工建设。广大市民期盼已久、投资近8亿元的火车站改扩建工程正抓紧设计。完成城市总体规划修编，到2020年城区规划面积将达到180平方公里。各县（市）积极行动，力度前所未有，一个大建设、大变样的局面正在展开。

过去的一年，我们倾力保障和改善民生，推动各项社会事业取得了新进展。高度重视和解决民生问题，投入56亿元，实施了40项惠民工程。新增城镇就业7.29万人，零就业家庭[3]保持动态为零。城镇登记失业率[4]低于省定目标。全年发放养老金、失业保险金32亿元，25万名离退休职工和失业人员的基本生活得到保障。34万人得到城乡最低生活保障。新农合参合率达到82.5％。城镇居民基本医疗保险全面启动，消除了我市医疗保险制度覆盖盲区。竣工101个乡镇卫生院，城市社区卫生服务覆盖面达到91%。城乡义务教育阶段公办学校学生学杂费全部免除。累计发放1.32亿元，资助贫困及优秀学生近10万人。近3000所农村中小学取暖设施全部改造完成，中央电视台焦点访谈给予报道。对5161户低收入住房困难家庭实施了住房保障。在燃煤价格成倍增长、供暖成本大幅提高的情况下，市财政补贴资金近7000万元，保持了供暖价格全省最低，减轻了市民负担。新解决200个村、20万人饮水安全问题，帮助5.1万贫困人口实现脱贫。市区新增绿地面积230万平方米，取缔、改造燃煤锅炉398台，新增集中供热面积200万平方米，空气质量二级以上天数增加7天，一级以上天数增加35天。深入开展亲民爱民活动，加强社会治安综合治理，促进了社会和谐稳定。“迎奥运、游保定”工作取得好成绩，国内旅游接待人数全省第一。科技、文化、计生、广播电视、民族宗教、妇女儿童、老龄、民兵、预备役等各项工作都取得了新成绩。特别值得提到的是，我省的3枚北京奥运金牌，都是保定籍运动员夺得的，他们为祖国争得了荣誉，为家乡人民争得了光彩！

各位代表，过去的一年，我们遇到的困难比估计的大，取得的成绩比预想的好。这是中共保定市委正确领导的结果，是市人大、市政协监督支持的结果，是全市1100万人民共同奋斗的结果。我代表保定市人民政府，向为保定发展做出无私奉献、付出辛勤劳动的全市人民，致以亲切问候！向给予政府工作大力支持的人大代表、政协委员，向老领导、老同志，向各民主党派、工商联、无党派人士和人民团体，向驻保人民解放军、武警官兵和各驻保单位，向所有关心、支持保定发展的港澳同胞、台湾同胞、海外侨胞、国内外朋友，表示诚挚感谢！在此，还要特别向在抗震救灾、奥运安保、处置三鹿奶粉事件工作中付出艰辛努力的医护人员、电力职工、建筑工人、政法公安干警、广大基层干部和志愿者，致以崇高敬意！

各位代表，需要特别报告的是，为兑现对人民代表的庄严承诺，市政府从2006年起每年都把《政府工作报告》的“话”落实到“事”上，把“事”落实到人上，实行“建账、查账、算账、报账”制度，分解立项，跟踪督办，推动落实。2008年《政府工作报告》共分解为486项具体事项，完成了462项，根据情况变化取消14项，由于主客观原因有10项没有完成。具体情况近日将通过媒体向社会公布。另外，由于奥运安保期间关停了一大批企业，金融危机又对我市汽车、纺织等行业造成冲击，尽管全市生产总值增速高于全省平均水平，但没有完成12.5%的预期目标。

各位代表，我们也清楚地认识到，全市经济社会发展和政府工作中还存在很多问题，还有许多事情应该做好没有做好、应该做到没有做到。从发展的深层次矛盾方面讲，工业化、城镇化发展水平低，经济总量小、人均水平低，发展方式转变不快、产业层次低，中心城市小、县域经济弱的问题依然突出。企业创新能力弱，市场发育水平低，推进改革开放的任务很重。环保压力大，节能减排、生态建设的任务还很艰巨。农村基础设施、社会公益事业方面还有很多欠账。社会保障能力差，就业压力很大。从经济社会管理工作方面讲，城市管理方式粗放，规划执行不严格，房地产市场管理不规范。马路市场、小街巷卫生死角、交通拥堵、“广告污染”、公交车覆盖面窄等问题依然突出。中小学校布局不合理、部分群众冬季取暖问题还没有很好地解决。一些医疗机构“过度医疗”，“小病当大病治”，检查多、药费贵的现象还比较严重。部分群众的合理诉求解决得还不够及时。食品、药品和生产安全监管还存在薄弱环节。社会治安状况还有待改善。从政府自身建设方面讲，思想解放不够，适应新形势、遵循市场经济规律抓发展的办法还不多，科学民主决策的水平还有待提高。职能转变还不到位，管理就是审批、执法就是罚款的现象依然突出。政府行政效能还不高，发展环境还不尽人意。少数公务员缺乏起码的职业道德，吃拿卡要，不作为、乱作为，企业和群众意见很大。个别工作人员奢侈浪费甚至以权谋私、贪污腐败，影响政府形象。对这些问题，我们一定继续高度重视，积极采取措施，认真加以解决。

二、2009年工作总体安排

今年可能是新世纪以来经济最困难的一年。当前，国际金融危机持续蔓延，世界经济增长明显减速，对我国经济的负面影响日益加深。从影响的广度和深度看，正从出口型城市向资源型城市、从沿海向内地逐步扩散，引发连锁反应，造成很多企业停工停产，就业岗位减少，收入水平降低，消费市场疲软，尤其对消费信心造成很大冲击。危机影响有多深、持续时间有多长，目前还难以估计。从对我市的直接影响看，汽车、纺织服装、房地产等行业受冲击较大，特别是中小企业面临很大困难，就业难问题更为突出。财政减收增支因素增多，收支矛盾加剧。这些新情况，与我市经济社会发展中存在的深层次矛盾叠加交织，加大了经济社会持续稳定发展的难度。面对这些挑战，我们必须把形势估计得严峻一些，把困难考虑得充分一些，把工作做得更扎实一些，做“最坏”的打算，争取最好的结果。

各位代表，我们也清楚地看到，在困难和挑战中蕴含着发展机遇和有利因素。从外部形势看，在经济危机影响下，国际国内资本寻求避风港是一种必然。我国经济加快发展的基本面没有改变，环渤海地区作为中国经济第三增长极加速崛起的态势没有改变，国际资本依然看好我国，尤其看好环渤海地区。我国成为世界制造工厂和南资北移、南企北上的步伐将继续加快。加之目前引进技术、人才的成本都在降低，正是我们打造投资热点的好时机。国家实施积极的财政政策、适度宽松的货币政策和一系列扩大内需政策，以及振兴汽车、纺织等产业的具体措施，为我们借力发展提供了难得机遇。我省把装备制造业作为继钢铁之后第二大产业来培育，也为我们做大“保定制造”创造了有利条件。从自身发展看，经过近年来持续“打基础、增后劲”，一批立市大项目陆续建成并产生效益，具备了较强的内生发展能力。新能源、绿色食品等主导产业在逆境中保持强势增长，以“中国电谷”为代表的新型产业优势开始显现。京石高铁、张石高速等重大基础设施项目相继开工建设，投资基础条件正发生根本性改善。京津冀一体化步伐加快，区位特点开始转化为发展优势。特别要看到，我们国家30年改革开放造就的抵御风险的强大实力和制度活力，我们党在危机面前统揽全局果敢决断的执政能力，我们民族不畏艰难自强不息的凝聚力和创造力。还要看到，在中共保定市委的坚强领导下，我市政治安定，团结和谐，正处在一个能干事、也能干成事的好时期。所有这些，为我们应对挑战、推进又好又快发展提供了有力保证。

历史经验表明，大危机往往蕴含大机遇，“大寒流”中也能做暖“小气候”。以做暖“小气候”应对冷环境，完全有可能逆势而上。只要我们继续咬定发展不放松，善用比较优势，勇抓相对机遇，努力把我们自己的事情办好，就一定能把外部不利影响降到最低，就一定能把好的发展势头保持下去。

今年政府工作的总体要求是：全面贯彻党的十七大和十七届三中全会精神，深入落实科学发展观，坚持加快发展、安全发展两手抓，狠抓项目建设保持经济较快增长，推进产业立市壮大经济实力，加强区域统筹培育发展新优势，深化改革开放增强发展活力和动力，注重社会建设加快解决民生问题，奋起直追、争先进位，努力推动又好又快发展、强市兴县富民，以优异成绩迎接新中国成立60周年。

全市经济社会发展的主要预期目标是：生产总值增长10%以上；万元生产总值综合能耗下降4.8%；主要污染物化学需氧量和二氧化硫排放量分别减少8%和4%；城镇固定资产投资增长22%；全部财政收入可比增长13%以上；地方一般预算收入可比增长13%以上；城镇居民人均可支配收入增长10%；农民人均纯收入增长8%；城镇登记失业率控制在4.6%以内；人口自然增长率控制在7.5‰以内。

今年政府工作的主要着力点是：保增长、保民生、保安全、保“变样”。保增长，就是面对严峻形势，始终把加快发展作为解决一切矛盾的根本，突出抓发展，重点抓项目，全力育产业，努力促进经济平稳较快增长。保民生，就是始终坚持以人为本，尽最大努力保障和改善民生。越是在经济困难的时候，越需要处理好财政拮据和保障民生的关系，越要把群众的冷暖安危放在首位，既做锦上添花的事情，更注重做雪中送炭的工作，集中财力解决就业、社会保障、扶弱济困等民生问题。政府要过紧日子，争取群众生活质量不下降。保安全，就是无论形势怎样变化，都必须把安全发展作为“底线”坚决守住。越是经济形势严峻的时期，越要坚持加快发展、安全发展两手抓，扎扎实实做好安全生产、食品药品安全、节能减排、社会稳定等各项工作，为加快发展创造安全稳定的社会环境。保“变样”，就是无论面临多大困难，都要坚定不移地推进“三年大变样”工作。发挥政府主导作用，遵循市场经济规律，引导全社会共同参与，以更大的决心、更强的力度、更高的水平推进各项建设，集中资源打造亮点，整合力量重点突破，确保城镇面貌取得让群众看得见的变化。

三、千方百计推进经济平稳较快增长

充分利用各种有利条件，发挥比较优势，挖掘增长潜力，集中要素资源，努力做强产业支撑，壮大经济实力。

培育壮大主导产业。抓住国家和省支持发展装备制造业的机遇，制定我市加快发展先进制造业规划，按照“南车北电东纺西绿城文”的产业格局，完善产业体系，放大产业优势，加快工业强市步伐。力促新能源产业快速增长。去年年底通过托管、共建[5]方式，“中国电谷”发展空间由14平方公里扩大到130平方公里。下一步，重点是制定完善发展规划，加快路、水、电、气等基础设施配套，推进六九硅业、中能风电等重大项目建设，继续扩大与兵装、国电等战略合作，加快扩张风电产业，做强光伏产业，壮大输变电、储电、节电等产业，努力建设世界一流的新能源产业基地。力促汽车工业稳步发展。用足用好国家汽车产业调整振兴规划和“微型车下乡”政策，继续坚持“差别竞争、错位发展”，支持企业扩张规模、完善链条、集群发展，推进华北轻型汽车城建设。推动中兴与长安、天马大迪与陕汽合作等项目取得实质性进展。抓好长城40万辆乘用车及零部件、河北长安30万台发动机和市汽车零部件基地等项目建设，千方百计帮助企业开拓市场。力促纺织服装产业转型升级。抓住国家出台纺织工业调整和振兴规划政策及北京建设“世界时装之都”的有利时机，推进与北京纺织服装产业战略合作，加快建设京南纺织服装生产加工基地。抓好园区建设和污染治理，确保中纺依棉精品工业园一期竣工投产，争取京棉公司7万纱锭整体搬迁项目落户宏润，加快高阳纺织印染循环经济园、白沟工业园、容城服装工业园等园区建设。做大绿色农副产品加工业。坚持引名企、育名牌、建基地，把农业生产优势转化为工业加工优势。突出抓好喜之郎、福满多等重点项目建设，争取江苏雨润集团项目落户我市，加快打造京津绿色农副产品加工供应基地。大力发展文化旅游产业。用好国家刺激旅游产业发展的政策机遇，培育一批“专、精、特、新”文化旅游企业，快上一批文化旅游项目，开发一批文化旅游产品，整合一批精品旅游线路，努力把资源优势转化为经济优势。突出抓好河北动漫产业基地、温泉城在水一方等重大项目，推进环京津休闲旅游产业带建设。发展壮大建筑建材业。立足优势，努力开拓国内外建筑市场。上大压小，重点抓好唐县冀东、曲阳金隅等大型干法水泥项目建设，扩大石材、新型建材生产规模，建设京南建材生产基地。鼓励电子信息等高新技术产业发展，努力打造新的产业支撑。

发展壮大县域经济。继续抓好园区经济、县城经济、民营经济和农村经济四个重点，全面提升县域经济发展水平。搞好园区建设。挖掘土地潜力，集中配置资源，每个县（市）至少规划建设一个有一定规模的工业园区。搞好环评和基础设施建设，引导企业向园区集中，打造县域经济发展平台。做强县城经济。抓好县城规划管理，引导资源要素向县城集中，统筹县城建设和产业布局，尽快把县城这个经济“龙头”做强。发展特色产业。对已经落伍的行业，要勇于放弃，自我革命，转型发展；对安国药业、白沟箱包、容城服装、高蠡纺织等传统产业，要调整结构，力促振兴；对涿州新材料、定州煤化工、安新铜业、徐水吊索具等新兴产业，要鼎力支持，尽快做大。壮大民营经济。以“全民创业活动”为载体，抓好融资担保、人才培训、技术研发等公共服务平台建设。各县（市）连续三年每年安排50亩土地，建设一批创业辅导基地。政府要积极营造环境，激发全民创业热情。

大力发展现代农业。越是在经济困难的时候，越要更加重视做好“三农”工作。全面贯彻落实党的十七届三中全会和中央一号文件精神，把各项新的支农惠农政策切实落到实处，夯实农业基础，发展农村经济，促进农民增收。稳定粮食生产，抓好“国家优质粮食产业工程”[6]和大型商品粮基地建设，力争连续第七年获得好收成。以乳品、肉类、粮油、果品、蔬菜五大优势农产品为重点，项目、基地、园区、产业整体推进，着力培育一批区域优势突出、特色鲜明的产业带。加快农业产业化进程，支持203家重点龙头企业加快发展。各县（市）力争每年引进1个投资1000万元以上的农业产业化项目。发展多种形式的农民专业合作组织，两年内覆盖农户60%以上。围绕种养、加工、销售等经营环节和种苗、技术推广、防疫等服务环节，加强政策支持和金融、科技、信息咨询服务，强化现代农业服务体系建设。推行标准化无公害生产，新建7个县级农产品综合质检站和13个动物产品检验检测中心，全面提高农产品质量安全水平。

加快发展服务业。把服务业作为拉动生产、促进消费的关键环节来抓。积极发展现代物流、金融保险、中介咨询、创意设计、服务外包、电子商务等生产性服务业，加快发展商贸、餐饮、娱乐、保健、家政等生活服务业，努力满足多层次消费需求。实施“万村千乡市场”[7]、“双百市场”[8]工程，做好“家电下乡”[9]工作，支持大型商业企业触角向农村延伸，积极开拓农村市场。结合“三年大变样”，加快“退二进三”，城镇黄金地段、金角银边优先用于服务业发展，培育一批新的服务业聚集区。把房地产业作为扩大投资、促进消费、保障民生的重要抓手，治理开发环境，提高审批效率，优化供应结构，落实优惠政策，鼓励购房消费，促进房地产业健康有序发展。

努力保持投资较快增长。坚持在快上项目中调优结构、在加大投资中推进增长。抢抓国家政策机遇，千方百计谋划、对接、争取项目。今年，安排市级重点项目250项，其中续建96项，开工74项，前期80项，总投资2400多亿元。积极做好规划、土地、环评等相关工作，更多地用改革的办法和市场机制破解融资、供地等难题，力求项目数量、质量和投资规模都有新突破。

四、统筹推进区域协调发展

把实施“一主三次”、“工业西进”、“对接京津”三大战略作为改变我市“小马拉大车”状况、推进城镇化、统筹区域发展的突破口，以新的城市总体规划为指导，培育发展新优势，活跃经济社会发展全局。

加快建设中心城市。以“三年大变样”为抓手，拆建并重，以点带面，全面推进。继续强力推进“五拆三改”，三年拆迁任务今年全部完成。重点抓好市区12条主干道、铁路沿线、7个出入口的环境治理，塑造城市新形象。开发改造府河、西大园、清真寺街3个片区和19个条件成熟的城中村，加快棚户区改建，建设宜居新区。基本完成光园、古城墙等古建筑修复和西大街、裕华路整治工程。开工建设火车站改扩建、关汉卿大剧院等一批标志性工程，确保电谷立交桥等10项精品工程国庆节前完工。加快建设“两环四廊、五湖十园”[10]城市绿化水系新景观。开放城市规划和建筑设计市场，完善规划设计决策机制，提高对城市景观风貌建设的控制力。发挥城建、土地、交通等五家投融资公司的作用，努力激活城市资源、资产，为城市建设提供有力的资金支撑。把今年作为城市精细化管理年，突出抓好背街小巷、高校、大型企业周边等重点部位环境整治，取缔马路市场，管好交通秩序，把群众身边环境治理好，打造干净有序文明的城市形象。

加快建设“一城三星一淀”大保定。按照新的城市总体规划，统筹市区和清苑、满城、徐水、安新四县规划建设。北部，通过扩张“中国电谷”空间，推动城区向北发展，加快与满城、徐水工业区对接；南部，依托长城、中兴和市汽车零部件基地向南拓展，加快与清苑对接；西部，畅通更多接山通道，加快与满城对接；东部，规划设计白洋淀大道和水运通道，快捷联通白洋淀。通过北扩、南拓、西接、东联，构建“南车北电强实力、西山东水映名城”的大城市格局。

加快建设“三次”中心城市。依托白沟·白洋淀温泉城，打造休闲旅游聚集区和京南商埠、陆港物流区。挖掘定州土地资源优势，建设定州唐河工业集中区，壮大产业基地。发挥涿州与北京接壤优势，发展高新技术产业和文化旅游等京郊服务业，建设现代化中等城市。以“三次”中心城市的率先崛起，打造新的区域经济增长极。

加快“工业西进”。这是改变西部山区贫困面貌、推进城乡统筹发展的重大战略。利用张石、保阜等高速公路交通条件，以工业园区为突破口，加快配套基础设施建设，引导工业企业向园区集中。发挥资源优势，做好资源对接资本、开发承接开放、产品带动产业三篇文章，谋划建设一批绿色产业、现代工业项目，以项目争取土地，以园区集聚项目。正确处理发展工业与保护环境的关系，严把园区规划环评、新上项目环保、县域节能减排总量控制“三个关口”，既要“金山银山”，更要“绿水青山”。

加快“对接京津”。环京津区位是我市最大的“资源”。在战略层面上，要把京津作为我市发展的参照系，把整个保定作为京津的大卫星城市来规划建设。当前，首先把毗邻京津的涞源、易县、涞水、涿州、高碑店、定兴、容城、安新、雄县、高阳等10个县（市）和白沟·白洋淀温泉城，作为率先融入京津的节点来抓，打造“环京津卫星城市链”。制定对接京津实施意见，统筹推进与京津发展规划、基础设施、产业、市场、人才、技术、文化等多层面对接合作，建设京津产业转移、科技成果转化、农副产品加工、休闲旅游和劳务输出五大基地。推进公交、通讯网络等市场一体化，培育“同城”思维，营造“同城”氛围，扩大“同城”效应，在融入京津中加快发展。

加强基础设施建设。构筑大交通。积极为京石高铁2012年通车创造建设条件，努力为保津城际铁路开工做好前期准备，构建铁路“十字交叉”枢纽。推进保阜、张涿、张石涞水至涞源段等5条(段) 高速公路建设，力争荣乌高速保定段、京石高速拓宽工程开工，加快张石高速北延至北京段前期工作。建设全国转体跨度最大的保阜高速跨京广铁路立交桥和华北地区最高的保阜高速黑崖沟特大桥。力争4年内高速公路新增600公里，总里程翻一番，建设“三纵三横一环”[11]京南高速公路枢纽港。抓好环城路建设，建设东三环、西二环，城南连通保阜路至张石段，城北连通徐水至张石段。推进白沟至温泉城快速路等4条（段）干线公路建设。抓好县乡村公路建设，两年内实现村村通客车。打造大水系。全面启动“两库连通、西水东调，引水济市、穿府补淀”工程。明年实现西大洋、王快两库连通。加快建设南水北调配套工程，尽快形成供水网络体系。加强电力设施建设。力争定洲电厂二期机组并网发电，保定南郊电厂、西北郊热电联产项目取得实质性进展。建设、改造220千伏以下电网4668公里，努力满足城乡用电需要。

五、努力保障和改善民生

在经济困难的时候，更要注重保障民生、注重社会建设。深入开展亲民爱民活动，全力帮助群众解决实际问题，让广大群众在和衷共济战胜困难中感受党和政府的关怀、社会大家庭的温暖。今年，重点实施10项民心工程办好62件实事。

扩大“创业型”社会就业。把就业再就业作为保民生的头等大事来抓。全面落实国务院做好高校毕业生就业和当前农民工工作的政策措施。搭建校企对接服务平台，组织举办就业洽谈会和专场招聘会，促进中等职业学校“订单培养”，鼓励大学生面向农村、面向中小企业就业。实施农村劳动力转移培训“阳光工程”[12]，开展回乡农民工大培训“春风行动”[13]，劳务输出45万人（次）以上。鼓励自谋职业、自主创业，从贷款发放、税费减免、工商登记、信息咨询等方面予以支持。积极开辟公共服务性岗位。加强对零就业家庭、失地农民等就业困难群体的重点帮扶。加强失业调控和失业预警，稳定社会就业总量，力争城镇新增就业7.5万人。尽最大努力帮助有就业愿望和就业能力的人找到工作。

加强“共享型”社会保障。健全养老、医疗、失业、工伤、生育等“五大社会保险体系”。抓好各类社会保险扩面。积极吸纳民营企业职工和农民工参保，包括在校大学生在内的城镇居民基本医疗保险参保率达到65%。提高企业退休人员待遇，确保各项社会保险金按时足额支付。完善最低生活保障、农村“五保”供养、大病医疗救助、教育助学、住房保障等“五大社会救助体系”。提高城乡低保对象生活补助标准，实施城乡低保金社会化发放。推进农村五保供养工作，集中供养率达到50%。加大医疗救助基金筹措力度，扩大医疗救助范围。筹集资金救助贫困学生9.43万人。加大住房保障力度，逐步建立以廉租住房为主、经济适用住房为辅的住房保障体系，尽快改善困难群众的居住条件。兴办“优抚”、“扶老”、“助残”、“救孤”、“济困”等“五大社会福利事业”。提高优抚对象生活补助标准。鼓励民间资本参与福利设施建设。探索日托式养老站。市儿童福利院竣工投入使用。救助特困残疾人5800名。发展社会慈善事业，抓好救灾救济和扶危济困工作，努力让广大群众共享发展成果。

发展“普惠型”社会事业。坚持教育优先发展。抓好素质教育，努力提高“一比两率”[14]，提高义务教育公用经费保障水平。加强规范化幼儿园和标准化学校建设，全市标准化小学、初中比例达到25%。加快普及高中阶段教育，改扩建25所高中，全市高中阶段毛入学率达到82%。推进中等职业教育和特殊教育学校建设，提高职业教育就业率。加快实施农村初中改造工程，完成农村中小学校舍维修改造6.5万平方米，努力把学校建成家长最放心、学生最安心的地方。加强公共卫生服务。认真落实国务院医改方案，促进基本公共卫生服务逐步均等化。加强乡镇卫生院建设，实施一批县、乡、村医疗卫生改扩建项目，实现农村医疗卫生服务全覆盖。新农合人均住院统筹封顶线由2万元提高到3万元。扩大城市社区卫生服务覆盖面，加大医疗惠民力度。深入开展医院管理年活动，严厉查处乱检查、乱加价、乱收费等问题，为群众就医提供更大方便。完善城市公共设施。市区新增公交车150辆，小型公交车辆进社区。加快改造社区支路和便民小路，方便市民出行。在市区繁华地段设置移动公厕，市区主次干道两侧单位厕所向市民免费开放。加快市区一户一表改造。谋划建设一批便民市场。实施“计生惠民”工程。落实农村计划生育家庭奖励扶助、特别扶助和“少生快富”等政策，加大社会抚养费征缴力度，严格人口与计划生育目标管理，稳定低生育水平，提高人口素质。发展文化事业。深入开展社会主义精神文明建设，加强社会公德、职业道德、家庭美德教育，倡树文明新风。加强文物管理与保护，完成全国第三次文物普查。深入开展文化下乡活动。加强文化基础设施建设，抓好乡镇综合文化站和“新农村书屋”[15]建设。开展新农村体育健身工程[16]和“百万农民健身活动”，举办戏剧节、空竹节、篮球节、敬老健身节等群众性文体活动，彰显“保定文化”特色，让群众生活更加丰富多彩。

进一步改善农民生产生活条件。加大对农村基础设施投入，抓好“路、水、电、气、房”五大工程，推进社会主义新农村建设。新改建农村公路900公里。解决245个村、29万人的饮水安全问题。加快实施西大洋、瀑河水库除险加固工程，启动21座中小水库的除险加固工程，整治行洪河道险工险段。抓好沙河、唐河、易水三大灌区节水改造。治理水土流失面积180平方公里。加强电气化县、乡、村建设和机井“户户持卡”改造。抓好秸秆固化气化试点和农村沼气池建设。深入推进扶贫开发，完成96个村的“整村推进”脱贫任务，稳定解决2.2万贫困人口的温饱问题。在搞好村庄规划的基础上，年内启动110个村的新民居建设，每个县(市、区)都要建设新民居样板村，让广大农民群众的居住条件尽快得到改善。

努力维护社会安全稳定。把今年作为“安全生产攻坚年”，强化政府监管责任，落实企业主体责任，加大执法、排查、整改力度，努力消除不安全隐患，坚决遏制重大安全事故。改革健全食品安全监管体制，整治食品生产经营秩序，逐步建立“从农田到餐桌”绿色供应体系和“从餐桌到农田”质量追溯机制，建设全国食品安全城市。加强社会管理，认真抓好矛盾纠纷排查，尽心尽力为群众解决实际问题。高度重视做好民族宗教工作，依法管理民族宗教事务。加强社会治安综合治理，严厉打击各种刑事犯罪活动，努力创造安全稳定的社会环境。

六、全面推进资源节约型、环境友好型社会建设

以建设“世界自然基金会低碳试点城市”[17]为抓手，推进节能减排，加强生态建设，加快形成节约能源和保护生态环境的产业结构、增长方式、消费模式，努力走出一条具有保定特色的生态文明发展之路。

打好节能减排攻坚战。全面推进省“双三十”和市“8+13”重点区域、重点企业节能减排工程进度，确保完成第二个年度任务。强化目标考核，对逾期未完成目标任务的县（市、区）和企业，严格执行问责制和“一票否决制”。开展“两厂（场）建设年和运行管理年”活动，确保7个县级垃圾处理场全部开工，在建的13个污水处理厂全部建成运行，已建成但未达到国家新标准的污水处理厂年内全部达标。加快污水管网收集系统建设，力争用两年时间实现城市（县城）污水全收集、全处理。建立重点河流跨县界断面水污染物排放监测和生态补偿机制，确保入淀河流水质稳定达标，努力保护好“华北明珠”白洋淀。

深入推进“蓝天行动”。启动天然气第二气源项目，加快建设集中供热网点，推行使用清洁能源，科学利用地热资源。加快“煤改气”进度，今年市区改造燃煤锅炉102台，减少燃煤4.78万吨，减排二氧化硫765吨。加快公交及出租车辆“油改气”，加强机动车尾气和建筑工地、环卫清扫二次扬尘污染治理，力保市区空气质量二级以上天数达到312天以上。

努力构筑城乡“绿色空间”。推进“太阳能之城”建设。推广绿色照明、新型绿色建筑材料应用，加快太阳能建筑一体化建设步伐。太阳能照明重点向小街巷、生活小区、公共场所和城镇延伸。到2010年，太阳能供热面积占到新增建筑面积的35%以上。实施“森林生态固碳计划”[18]，加快城乡绿化步伐，完成造林50万亩，义务植树1500万株，全市森林覆盖率达到23%。广泛开展生态县(市)、环境优美城镇、环境友好企业等创建活动，争创国家园林城市和国家森林城市。

大力倡导低碳生产生活方式。认真落实《关于建设低碳城市的意见》，以节约能源、资源为核心，加快关键领域低碳技术开发应用，减少煤炭、石油等化石能源消耗。发展低碳产业，倡导低碳生活，建设低碳社会。抓好一批清洁生产试点企业，发展“静脉产业”[19]。广泛开展低碳机关、社区、学校、家庭、宾馆创建活动，引导人们在衣、食、住、行等日常生活中节约能源资源，建设生态文明的绿色保定。

七、大力推进改革开放和科技创新

深化改革、扩大开放、科技创新，是保增长、促发展的三大动力，必须加大力度，突破体制机制障碍，增创发展新优势。

加快推进各项改革。一是抓好市对县、市对区管理体制改革。总的原则是放权松绑、重心下移、激发活力。全面落实《关于向县（市）下放若干管理权限的意见》，把项目、土地、环保等部分审批管理权限下放到县（市）。坚持统放结合，责权利相统一，逐步把城市管理权限下放到区。二是推进国有企业改革。全面完成市县属中小企业改制。推动河北钢铁集团对涞钢、涞铜战略重组。加快亏困企业改制步伐。推进城市公用事业单位改革。三是推进农村综合改革。落实最严格的耕地保护制度和最严格的节约用地制度。以农民增收为目的，推进农村土地依法、自愿、有偿流转。抓好集体林权制度改革。放宽城市、放开县城户口迁移政策，为农民进城打开“绿灯”。四是推进财税体制改革。实施市对县激励性财政体制，壮大县级财政实力。落实增值税转型改革，减轻企业负担。深化预算管理、政府采购、资本收益、财政监管等制度改革，推行政府投资非经营性项目代建制，认真把纳税人的钱管好、用好。五是推进金融体制改革。从优化金融环境、搭建融资平台、引进金融机构入手，努力做强金融支撑。抓好市商业银行二次增资扩股、延伸分支机构工作。推进农村信用社向农村合作银行、商业银行发展，搞好村镇银行、小额贷款公司等新型金融机构试点工作。努力构建政、银、企“零距离”对接的融资机制。

进一步扩大对外开放。密切关注资本流动新趋势，抓好宣传推介，瞄准国内外知名公司和龙头企业，充分发挥三个专业招商中心的作用，大力开展园区招商、产业招商，力争引资质量和规模实现新突破，在全方位开放中提速发展。做好巨力、中航惠腾、味群3家企业的上市培育工作。实施科技兴贸和品牌兴贸战略，落实国家新能源出口创新基地、国家汽车及零部件出口基地支持政策，积极开拓新兴国际市场，推进出口市场多元化。鼓励企业“走出去”。

努力提高科技创新能力。以建设创新型保定为核心，健全以市场为导向、企业为主体、产学研相结合的技术创新体系。重点支持企业研发中心建设，组织开展新能源、汽车、纺织、中药等行业关键技术攻关，实施一批农村科技支撑项目，加快科技成果转化应用。按市场机制组建保定市科技风险投资公司，促进高科技产业快速发展。深入实施人才强市战略，用好驻保高校资源，抓好校企合作，发展“大学经济”。积极引进一批高层次创新创业人才，培养一批高科技人才，让更多有才之士在保定成长、在保定成功。

八、进一步加强政府自身建设

复杂多变的形势、繁重艰巨的任务，对政府自身建设提出了新的更高要求。特殊时期要有非常作为。各级政府工作人员必须以更高标准履行职责、勤奋工作，以良好的精神状态迎接挑战。

特殊时期，更需要把握大势、科学决策。面对复杂的经济形势，要更加注重学习，加强前瞻性研究，善用“大视野”审视“小环境”，用战略思维研究战术问题，在危机中寻求机遇，在逆境中探索出路，增强在复杂局面下推进工作的能力。面对艰巨的发展任务，要更加注重调查研究，问政于民、问需于民、问计于民，避免走弯路和“建设性破坏”。面对前进中的困难，要更加注重妥善处理好各方面的利益关系，着力解决制约发展与影响稳定的关键问题，把握全局，突出重点，努力掌握加快发展、安全发展的主动权。

特殊时期，更需要转变作风、改善环境。越是经济困难时期，越要改善“软环境”，应对“硬挑战”。今年，在政府系统深入开展“作风建设年”活动。突出治庸、治懒、治梗、治乱，着力解决不尽职责、服务差，不讲标准、效率低，不守纪律、乱作为等问题。对少数危害发展环境的害群之马，一经查实，坚决清理出公务员队伍。规范行政审批，压缩审批时限，削减审批事项。严格行政执法，坚决查处乱检查、乱收费行为，切实保障企业合法权益。实行服务承诺制、首问首办责任制、限时办结制，提高行政效能。实行服务评价和工作满意度投票制度，努力在服务中创新管理、在管理中体现服务。加快电子政务建设，年内完成电子审批网络和电子监察系统建设，实现行政许可项目在线审批。推进市行政服务中心“二次改革”，按照“两集中、两到位”[20]要求，整合机构设置，加大进厅力度，宁可让机关“伤筋动骨”，也不能让群众和企业费力伤神！

特殊时期，更需要埋头苦干、狠抓落实。各级政府工作人员必须紧张起来，把全部心思用在抓发展、保安全上。不讲客观条件、多做主观努力，定下来的事雷厉风行，看准了的事一抓到底。坚持制度管事，管事务必从严。严格落实县域经济省内综合排位、重点项目建设、节能减排、安全生产、“三年大变样”等“红黄牌”警示制度，加强绩效考核，努力增强执行力。对今年的《政府工作报告》，还要继续分解立项制定《落实方案》，向社会公布，接受监督，不折不扣兑现对人民的承诺。

特殊时期，更需要艰苦奋斗、勤政廉洁。面对今年财政困难的局面，必须树立过紧日子的思想。各级政府要带头，勤俭办一切事业，节约办一切活动。严格控制行政开支，强化预算约束。今年公务购车、会议经费、公务接待费用以及出国（境）经费等支出实行“零增长”。深入开展行政监察和审计监督，严厉查处领导干部滥用职权、贪污贿赂、失职渎职案件，对腐败分子坚决依法惩处、绝不容情。

各位代表，我们深知，政府的一切权力都是人民给的，政府工作必须置于广大人民的监督之下。我们一定继续推进权力公开透明运行，自觉接受人大及其常委会法律监督和工作监督，自觉接受政协民主监督、人民群众监督和舆论监督，认真听取各民主党派、工商联、离退休老干部和社会各界人士的建议和意见，主动加强与工会、妇联、共青团等人民团体联系，不断改进政府工作，履行好政府职责，不辜负全市人民的信任和期望。

各位代表，团结凝聚力量，奋斗铸就辉煌。我们坚信，任何困难都改变不了保定人民创造美好生活的强烈愿望，任何挑战都阻挡不了我们加快发展的坚定步伐！让我们更加紧密地团结在以胡锦涛同志为总书记的党中央周围，在省委、省政府和中共保定市委的坚强领导下，深入贯彻落实科学发展观，齐心协力，克难奋进，为推动又好又快发展、强市兴县富民而努力奋斗！
